# Linkage disequilibrium, persistence of phase and effective population size estimates in Hereford and Braford cattle

**DOI:** 10.1186/s12863-016-0339-8

**Published:** 2016-02-01

**Authors:** Patrícia Biegelmeyer, Claudia C. Gulias-Gomes, Alexandre R. Caetano, Juan P. Steibel, Fernando F. Cardoso

**Affiliations:** Programa de Pós-Graduação em Zootecnia, Faculdade de Agronomia Eliseu Maciel, Universidade Federal de Pelotas, Capão do Leão, Rio Grande do Sul Brazil; Embrapa Pecuária Sul, Bagé, Rio Grande do Sul Brazil; Embrapa Recursos Genéticos e Biotecnologia, Brasília, Distrito Federal Brazil; Programa de Pós-Graduação em Ciências Animais, Faculdade de Agronomia e Medicina Veterinária, Universidade de Brasília, Brasília, Distrito Federal Brazil; Conselho Nacional de Desenvolvimento Científico e Tecnológico (CNPq), Brasília, Distrito Federal Brazil; Michigan State University, East Lansing, MI USA

**Keywords:** Beef cattle, Genetic diversity, Linkage disequilibrium, Quantitative trait loci, Single nucleotide polymorphism

## Abstract

**Background:**

The existence of moderate to high levels of linkage disequilibrium (LD) between genetic markers and quantitative trait loci (QTL) affecting traits of interest is fundamental for the success of genome-wide association (GWAS) and genomic selection (GS) studies. Knowledge about the extent and the pattern of LD in livestock populations is essential to determine the density of single nucleotide polymorphisms (SNP) required for accurate GWAS and GS. Moreover, observed LD is related to historical effective population sizes (*N*_*e*_), and can provide insights into the genetic diversity history of populations. Estimates of the consistency of linkage phase across breeds (*R*_*H,B*_) can be used to determine if there is sufficient relationship to use pooled reference populations in multi-breed GS programs. The objective of this study was to estimate LD levels, persistence of phase and effective population size in Hereford and Braford cattle populations sampled in Brazil.

**Results:**

Mean LD estimates, measured using the squared correlation of alleles at two loci (*r*^*2*^), obtained between adjacent SNP across all chromosomes were 0.21 ± 0.27 for Herefords (391 samples with 41,241 SNP) and 0.16 ± 0.22 for Brafords (2044 samples and 41,207 SNP). Estimated *r*^*2*^ was > 0.2 and 0.3, respectively, for 34 and 25 % of adjacent markers in Herefords, and 26 and 17 % in Brafords. Estimated *N*_*e*_ for Brafords and Herefords at the current generation was 220 and 153 individuals, respectively. The two breeds demonstrated moderate to strong persistence of phase at all distances (*R*_*H,B*_ = 0.53 to 0.97). The largest phase correlations were found in the 0 to 50 Kb bins (*R*_*H,B*_ = 0.92 to 0.97). Estimated LD decreased rapidly with increasing distance between SNP, however, useful linkage for GWAS and GS (*r*^*2*^ > 0.2) was found spanning to ~50 Kb.

**Conclusions:**

Panels containing about 50,000 and 150,000 SNP markers are necessary to detect minimal levels of LD between adjacent markers that would be useful for GWAS and GS studies to Hereford and Braford breeds, respectively. Markers are expected to be linked to the same QTL alleles in distances < 50 Kb in both populations due to observed high persistence of phase levels.

## Background

The evolution of molecular biology tools and techniques occurred over the last decades helped unveiling underlying genetic factors and improving rates of genetic gains for economically important traits in livestock. The availability of high-density single nucleotide polymorphisms (SNP) genotyping assays for cattle coupled with advances in computational and statistical methods allowed the generation and use of large amounts of genomic data in genome-wide association (GWAS) and genomic selection (GS) studies for production-relevant traits [1]. These advancements created new opportunities to identify and select animals with superior genetic merit, while reducing generation intervals and overall associated costs [2, 3].

Existing genome-wide linkage disequilibrium (LD) levels are usually affected by genetic (selection, mutation, drift, migration, and non-random mating) and non-genetic (marker ascertainment bias) factors [4–6] and they can reflect historical effective population sizes and rates of recombination in a population [7]. Studies to understand LD levels and structure in livestock species are necessary for revealing diversity levels among breeds and detecting regions of genome that have been historically subjected to different selection pressures [8, 9]. Knowledge about historical effective population sizes is also important to determine optimal selection pressures [10] for achieving breeding goals while maintaining acceptable levels of genetic diversity in breeding populations [11]. Estimates of linkage phase consistency across breeds and populations are also essential for determining the potential success of using data from pooled reference populations for multi-breed genomic evaluation and selection programs [12].

Hereford and Braford cattle are important breeds for beef production in southern Brazil, where subtropical climates are observed with average low temperatures in winter months. Local climate conditions naturally control the incidence of tropical ectoparasites such as the bovine tick (*Rhipicephalus (Boophilus) microplus*) however, infestation peaks can be observed sporadically, raising risks of losses associated with tick-borne diseases [13–15]. Development of strategies to implement GS methods for genetic improvement of tick-resistance in these breeds are underway [16], in addition to studies focusing on unrevealing biological mechanisms underlying this trait [17]. Minimal levels of linkage disequilibrium between causative variants and genetic markers are fundamental for performing effective GWAS and GS studies, since these approaches rely on the non-random associations between markers and functional mutations affecting traits of interest [18, 19]. Successful experimental designs for performing GWAS and GS with Brazilian Hereford and Braford cattle will be highly dependent of the right choice of marker density, which in term is dependent on the estimated levels of LD across the genome [20].

The objective of this study was to estimate linkage disequilibrium levels at varying SNP densities, persistence of phase and effective population sizes in Brazilian Hereford and Braford cattle populations.

## Methods

### Animal welfare

All experimental procedures involving live animals were approved by the Committee for Ethics in Animal Experimentation from the Federal University of Pelotas (Comissão de Ética em Experimentação Animal, Pelotas, Rio Grande do Sul, Brazil; process number 6849).

### Sample collection and genotyping

Samples from a total of 391 Hereford and 2079 Braford (with a breed composition ranging between ½ Hereford + ½ Zebu and ¾ Hereford + ¼ Zebu) cattle born between 2008 and 2010 in commercial farms associated with the Delta G Connection Genetic Improvement Consortium [21] were used in this study. DNA was extracted from blood samples from FTA cards or from cryopreserved semen. Genotyping of all samples was performed with Illumina BovineSNP50v2 (50 K) BeadChip (Illumina Inc., San Diego, USA). Additionally, data from 40 bulls (17 Hereford and 23 Braford) genotyped with Illumina High-Density (HD) Bovine BeadChip Array (Illumina Inc., San Diego, USA) were also included in the final dataset. Genotype calling and initial data quality control (QC) were performed using GenomeStudio software (Illumina Inc. San Diego, CA), according to manufacturer's protocols. Genotypes with a GenCall Score < 0.15 were set as missing genotypes.

### Data quality control

Additional QC was performed with R snpStats package [22, 23]. Samples with call rates < 0.90, heterozygosity deviations > 3.0 standard deviations, conflicts between declared and genotype-based sex, and duplicated genotypes with different sample identification were removed from the final dataset. Only SNP located on autosomes (BTA) were considered in further analyses. SNP with call rates < 0.98, minor allele frequencies (MAF) < 0.03 and highly significant deviations (*P* < 10^-6^) from Hardy-Weinberg Equilibrium (HWE) were also excluded. Moreover, whenever multiple SNP were observed in the same physical map position, only one SNP with highest MAF was retained. The high-density (HD) panel was filtered to select only the SNP also present in the 50K panel.

### Haplotype reconstruction and phasing

Haplotype reconstruction and imputation of sporadically missing genotypes (0.39 %) were carried out using FImpute 2.0 [24]. Expectation-Maximization algorithm employed initially estimates the most probable haplotypes considering observed genotypes, using pedigree relationship information. Subsequently, the program performs genotype imputation using a haplotype search based on a sliding window approach, walking along each chromosome and using overlapping windows to reconstruct haplotypes [25].

### Linkage disequilibrium analysis

Linkage disequilibrium was calculated as pairwise *r*^*2*^ [26], which relies on the allele phase information at the gametic level. Considering two marker loci (A and B), each one with two alleles (A1, A2, B1 and B2), the frequency of alleles in the population can be denoted *p*_A1_, *p*_A2_, *p*_B1_ and *p*_B2_, and the frequency of haplotypes with allele 1 at marker locus A and allele 1 at locus B, for example, denoted *p*_A1B1_. Following Hill and Roberson [26],

1 $$ {\mathtt{r}}^{\mathtt{2}}=\frac{{\left({\mathtt{p}}_{\boldsymbol{A1}\boldsymbol{B1}}{\mathtt{p}}_{\boldsymbol{A2}\boldsymbol{B2}}\hbox{-} {\mathtt{p}}_{\mathrm{A}1\mathrm{B}2}{\mathtt{p}}_{\mathrm{A}2\mathrm{B}1}\right)}^{\boldsymbol{2}}}{{\mathtt{p}}_{\boldsymbol{A1}}{\mathtt{p}}_{\boldsymbol{A2}}{\mathtt{p}}_{\boldsymbol{B1}}{\mathtt{p}}_{\boldsymbol{B2}}}. $$

For each population, LD values between all pairs of SNP of all chromosomes were binned according to pairwise physical distances into intervals of 100 Kb starting from 0 up to 10 Mb. Average values of *r*^*2*^ were calculated for each bin.

To evaluate the feasibility of successfully using sparser marker panels in GWAS and GS studies, average *r*^*2*^ between adjacent markers was calculated for different marker densities, sequentially removing SNP from the total dataset using every second, fourth, fifth, sixth, seventh and 14^th^ marker (using, respectively, 50, 25, 20, 17, 14 and 7 % of available SNP). Linkage disequilibrium estimates were calculated according to Badke et al. [27], using R scripts [23] available at https://www.msu.edu/~steibelj/JP_files/LD_estimate.html.

### Intra and inter chromosomal and breed heterogeneities

To investigate intra and inter chromosomal and breed variation in LD, two analysis of covariance with general linear models were fitted. The following linear model (Equation 2) was used to estimate the effects of physical distance (covariate), chromosome, breed and the chromosome x breed interaction on LD through a total of 82,356 adjacent breed-specific marker pairs:

2 $$ {\mathtt{r}}_{\mathtt{i}\mathtt{j}\mathtt{k}}^{\mathtt{2}}=\mathtt{\mu}+{\mathtt{c}}_{\mathtt{i}}+{\mathtt{b}}_{\mathtt{j}}+{\mathtt{\beta}}_{\boldsymbol{1}}\left({\boldsymbol{d}}_{\mathtt{k}}^{*}\right)+\mathtt{c}{\mathtt{b}}_{\mathtt{i}\mathtt{j}}+{\mathtt{e}}_{\mathtt{i}\mathtt{j}\mathtt{k}}, $$

were ${\mathtt{r}}_{\mathtt{ijk}}^{\mathtt{2}}$ was the observed LD over marker distance ${\mathrm{d}}_{\mathit{\mathsf{k}}}$ on chromosome *i* of breed *j*, *μ* is the overall mean of ${\mathtt{r}}_{\mathtt{ijk}}^{\mathtt{2}}$ across markers pairs, ${\mathtt{c}}_{\mathtt{i}}$ is the effect of chromosome *i*, ${\mathtt{b}}_{\mathtt{j}}$ is the effect of breed *j*, ${\mathtt{\beta}}_{\mathsf{1}}$ is the regression coefficient on marker distance, ${\boldsymbol{d}}_{\mathtt{k}}^{*}$ is the adjusted marker distance $\left(\boldsymbol{l}\boldsymbol{o}{\boldsymbol{g}}_{\boldsymbol{10}}{\boldsymbol{d}}_{\boldsymbol{k}}-\boldsymbol{l}\boldsymbol{o}{\boldsymbol{g}}_{\boldsymbol{10}}\overline{\boldsymbol{d}}\right)$, ***d***_***k***_ is the observed physical distance for marker pair *k*, $\overline{\boldsymbol{d}}$ is the average physical distance between markers, and ${\mathtt{e}}_{\mathtt{ijk}}$ is the residual effect. Distances were log_10_ transformed in an attempt to linearize the relationship between LD and the log-transformed distance [28].

Additionally, a more complex linear model was fit to all 12,911,174 syntenic SNP pairs to investigate the adjusted mean *r*^*2*^ in a broader range of inter marker distances than that observed in Equation 2, where just adjacent markers were considered. This more comprehensive model can be represented by:

3 $$ \begin{array}{c}{\mathtt{r}}_{\mathtt{i}\mathtt{j}\mathtt{k}}^{\mathtt{2}}=\mathtt{\mu}+{\mathtt{c}}_{\mathtt{i}}+{\mathtt{b}}_{\mathtt{j}}+{\mathtt{\beta}}_{\mathsf{1}}\left({\boldsymbol{d}}_{\mathtt{k}}^{*}\right)+\mathtt{c}{\mathtt{b}}_{\mathtt{i}\mathtt{j}}+\mathtt{c}{\mathtt{\beta}}_{\mathsf{1}\mathtt{i}}\left({\boldsymbol{d}}_{\mathtt{k}}^{*}\right)+\mathtt{b}{\mathtt{\beta}}_{\mathsf{1}\mathtt{j}}\left({\boldsymbol{d}}_{\mathtt{k}}^{*}\right)+\mathtt{c}\mathtt{b}{\mathtt{\beta}}_{\mathsf{1}\mathtt{i}\mathtt{j}}\left({\boldsymbol{d}}_{\mathtt{k}}^{*}\right)+{\mathtt{\beta}}_{\mathsf{2}}{\left({\boldsymbol{d}}_{\mathtt{k}}^{*}\right)}^{\boldsymbol{2}}+\mathtt{c}{\mathtt{\beta}}_{\mathsf{2}\mathtt{i}}{\left({\boldsymbol{d}}_{\mathtt{k}}^{*}\right)}^{\boldsymbol{2}}+\\ {}\mathtt{b}{\mathtt{\beta}}_{\mathsf{2}\mathtt{j}}{\left({\boldsymbol{d}}_{\mathtt{k}}^{*}\right)}^{\boldsymbol{2}}+\mathtt{c}\mathtt{b}{\mathtt{\beta}}_{\mathsf{2}\mathtt{i}\mathtt{j}}{\left({\boldsymbol{d}}_{\mathtt{k}}^{*}\right)}^{\boldsymbol{2}}+{\beta}_{\mathsf{3}}{\left({\boldsymbol{d}}_{\mathtt{k}}^{*}\right)}^{\boldsymbol{3}}+\mathtt{c}{\mathtt{\beta}}_{\mathsf{3}\mathtt{i}}{\left({\boldsymbol{d}}_{\mathtt{k}}^{*}\right)}^{\boldsymbol{3}}+\mathtt{b}{\mathtt{\beta}}_{\mathsf{3}\mathtt{j}}{\left({\boldsymbol{d}}_{\mathtt{k}}^{*}\right)}^{\boldsymbol{3}}+\mathtt{c}\mathtt{b}{\mathtt{\beta}}_{\mathsf{3}\mathtt{i}\mathtt{j}}{\left({\boldsymbol{d}}_{\mathtt{k}}^{*}\right)}^{\boldsymbol{3}}+{\mathtt{e}}_{\mathtt{i}\mathtt{j}\mathtt{k}}.\end{array} $$

In addition to the variables already described above for Equation 2, here ${\mathtt{\beta}}_{\mathsf{2}}$ and ${\mathtt{\beta}}_{\mathsf{3}}$ are the regression coefficients (quadratic and cubic, respectively) on marker distance. Although the log_10_ transformation of physical distances to linearize the relationship between LD and the log-transformed distances, we decided to fit these higher order coefficients to consider possible deviations of the expected linearity and interaction with breed and chromosome effects (*cβ*_1*i*_, *cβ*_2*i*_, *cβ*_3*i*_, *bβ*_1*j*_, *bβ*_2*j*_, *bβ*_3*j*_, *cbβ*_1*ij*_, *cbβ*_2*ij*_ and *cbβ*_3*ij*_ terms).

The physical distances of interest to predict *r*^*2*^ using Equation 3 were related to the average inter marker distance values observed in some commercial panels available for cattle genomic selection. The density of the considered panels was 150K (GeneSeek Genomic Profiler HD-150K), 80K (GeneSeek Genomic Profiler HD-80K), 50K (Illumina BovineSNP50v2 BeadChip), 20K (GeneSeek Genomic Profiler LD v2), 8K (Illumina BovineLD v.2 BeadChip) and 3K (Illumina Bovine3k BeadChip). Assuming a bovine genome size of 3.000 Mb [29], the target distance values were calculated dividing the genome length in Mb by the number of markers in each panel. As example, for the 150K panel, we divided 3.000 Mb by 150.000 markers and obtained an average inter marker distance (d_k_) of 20 Kb. Accordingly, chromosome by breed predicted *r*^*2*^ values were obtained from estimated parameters of Equation 3 at physical inter marker distances of 20, 38, 60, 150, 375 and 1000Kb. All analysis was performed using R package [23].

### Analysis of persistence of phase

The degree of phase concordance between the two breeds for pairs of SNP was calculated according to Badke et al. [27] with the following formula:

4 $$ {R}_{B,H}=\frac{{\displaystyle \sum_{\left(i,j\right)\in p}\left({r}_{ij(B)}\hbox{-} {\overline{r}}_{(B)}\right)\left({r}_{ij(H)}\hbox{-} {\overline{r}}_{(H)}\right)}}{S_{(B)}{S}_{(H)}}, $$

where *R*_*B*,*H*_ is the correlation of phase between *r*_*ij*(*B*)_ in the Braford (*B*) population and *r*_*ij*(*H*)_ in the Hereford (*H*) population, *S*_(*B*)_ and *S*_(*H*)_ are the standard deviations of *r*_*ij*(*B*)_ and *r*_*ij*(*H*)_, respectively, ${\overline{r}}_{(B)}$ and ${\overline{r}}_{(H)}$ are the average *r*_*ij*_ across all SNP *i* and *j* within interval *p* for populations *B* and *H*, respectively.

Positive *r* values are expected when two markers are in LD and show equal linkage phase in both studied populations [30]. Marker pairs were binned according to marker distances (intervals of 100 Kb starting from 0 up to 10 Mb), and average values of *R*_*B*,*H*_ were calculated for each bin, using markers common to both breeds.

### Estimation of effective population size

Estimates of LD decay in relation to different SNP distances were used to infer past effective population sizes (*N*_*e*_) of the two studied cattle breeds. The relationship between *r*^*2*^ and *N*_*e*_ can be expressed by the formula:

5 $$ E\left({r}^2\right)=\frac{1}{\left(4c{N}_e+1\right)}, $$

where *c* is the genetic distance between two markers expressed in Morgans [31]. Effective population size was estimated considering each SNP pair located within 100 Mb of the same chromosome, with physical distances between SNP converted to genetic distances, assuming 1 Mb = 1 cM [32, 33]. Because generations are discrete and distances between SNP are continuous, historical effective population size (*N*_*et*_) for a given generation *t* = 1/2*c* [34] in the past was assessed by selecting SNP pairs with map distance within corresponding ranges of *c* values. When applied in *t* = 1/2*c,* the resulting *t* value was rounded to the target generation. For example, *r*^*2*^ of all SNP pairs with distance between 33.3 cM (*t* = 1.5) and 1 M (*t* = 0.5) of the same autosomal chromosome were selected to calculate *N*_*e*_ at *t* = 1. Then, within each bin, the average values of *r*^*2*^ and *c* were obtained and applied in the formula:

6 $$ {N}_{et}=\frac{\left(1\hbox{-} {r}^2\right)}{4c{r}^2}, $$

for 0.0 < *r*^*2*^ < 1.0. Longer *c* ranges were used to define generation bins as we moved further in the past, because they correspond to shorter distances with fewer markers and we wanted to ensure sufficient numbers of SNP pairs for reliably estimating *N*_*et*_ within each bin [35]. The actual bins were of one generation for *t* between 1 and 10, e.g. a range from 0.5 to 1.5 for the current generation; five generations for *t* between 15 and 100, and of 50 generations for *t* between 150 and 1000 (see Table 1 for additional details).

**Table 1:** Description of generation binning process

Generation range applied to	Number of generations represented by each bin	Example for first bin		
		Generation	Generation range	Corresponding distance range [mid-point] (Morgans)
1–10	1	1	0.5 to 1.5	0.33 to 1.0 [6.7 × 10^-1^]
15–100	5	15	12.5 to 17.5	0.04 to 0.02857 [3.33 × 10^-2^]
150–1000	50	150	125 to 175	0.004 to 0.00286 [3.33 × 10^-3^]

## Results

### SNP quality control

Data QC excluded 43 samples with call rates < 0.90, 24 samples with heterozygosity deviations > 3.0 standard deviations, eight samples with incorrect sex assignment and eight duplicated genotypes with different sample identification. The final resulting dataset contained 2435 samples (391 Hereford and 2044 Braford). Some samples were excluded for not meeting more than one criteria of QC. Marker QC also removed 4232 SNP with call rates < 0.98, 5712 SNP with MAF < 0.03, and 1342 SNP with highly significant HWE deviations (*P* < 10^-6^). Estimated means for call rate, MAF and HWE were 0.998, 0.271, and 0.541, respectively. Additionally, 34 monomorphic markers in the subset of Hereford samples were excluded from subsequent analyses of this breed. The final resulting dataset contained a total of 41,241 autosomal SNP (75.52 %) in Brafords and 41,207 autosomal SNP (75.46 %) in Herefords.

### Linkage disequilibrium

**Fig. 1:**
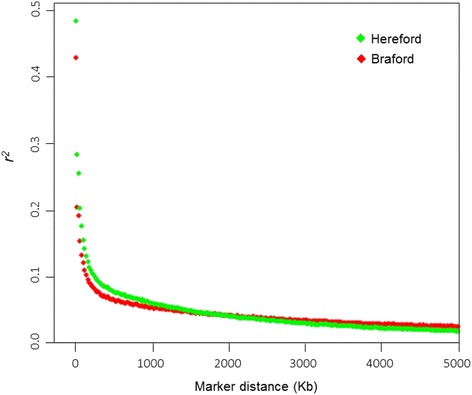
Extent of *r* ^*2*^ as a function of inter-marker distance in Hereford and Braford populations

Average *r*^*2*^ ± SD between adjacent SNP across all chromosomes was 0.21 ± 0.27 for Hereford and 0.16 ± 0.22 for Braford. The analysis revealed a rapid decrease in LD in both populations with increasing physical distances (Fig. 1). Herefords showed higher LD than Brafords up to a distance of 1780 Kb. For larger distances, Brafords showed a mean of *r*^*2*^ slightly higher than Herefords. Average LD for markers at some distance intervals is presented in Table 2. At distances of 50 Kb, average LD was 0.25 ± 0.29 for Herefords and 0.18 ± 0.24 for Brafords. In Brafords, average *r*^*2*^ decayed faster than in Herefords with the increase of distance, declining to 50 % of its initial value at ~5 Kb whereas in Herefords the same decline was observed at ~50 Kb. Observed LD was > 0.2 and 0.3, respectively, for 34 % (14,010 SNP) and 25 % (10,302 SNP) of adjacent markers in Herefords, and 26 % (10,722 SNP) and 17 % (7011 SNP) in Brafords.

**Table 2:** Average *r* ^*2*^ ± SD between adjacent markers according to inter-marker distances

Inter-marker distance	Hereford	Braford
0–1 Kb	0.49 ± 0.45	0.43 ± 0.39
1–5 Kb	0.28 ± 0.35	0.20 ± 0.30
0–20 Kb	0.29 ± 0.34	0.21 ± 0.27
20–40 Kb	0.26 ± 0.30	0.19 ± 0.24
5–50 Kb	0.25 ± 0.29	0.18 ± 0.24
40–60 Kb	0.20 ± 0.26	0.15 ± 0.21
60–80 Kb	0.18 ± 0.23	0.13 ± 0.19
50–100 Kb	0.17 ± 0.23	0.13 ± 0.18
0.1–0.5 Mb	0.10 ± 0.15	0.08 ± 0.12
0.5–1 Mb	0.07 ± 0.11	0.06 ± 0.09
2–5 Mb	0.03 ± 0.05	0.03 ± 0.05
5–10 Mb	0.01 ± 0.02	0.02 ± 0.03

Estimated *r*^*2*^ values > 0.2 were observed in the 0 to 60 Kb bins in Herefords (range = 0.20 to 0.49), and in the 0 to 20 Kb bins in Brafords (range = 0.21 to 0.43). Average *r*^*2*^ values > 0.3 were observed in the 0 to 1 Kb bins in Herefords (0.49) and Brafords (0.43). Linkage disequilibrium estimates obtained from sparse maps of markers were low (Table 3). Considering only 50 % of available SNP (a panel with about 20K SNP), observed average *r*^*2*^ decreased from 0.21 to 0.15 in Herefords, and from 0.16 to 0.12 in Brafords. When 20 % of SNP were maintained in the map (using only every fifth SNP and a density similar to the 8K panel), average *r*^*2*^ decreased to 0.09 in Herefords and to 0.08 in Brafords. Similarly, as observed in LD values, the percentage of pairs of markers with *r*^*2*^ values > 0.2 and with average *r*^*2*^ > 0.3 decreased when sparse maps were analyzed (Table 4). Using only every fifth marker (20 % of available SNP), the percentage of markers with *r*^*2*^ > 0.2 decreased from 34 to 15 % in Herefords and from 26 to 10.1 % in Brafords, while the percentage of markers with *r*^*2*^ > 0.3 decreased from 25 to 8.3 % in Herefords, and from 17 to 4.9 % in Brafords.

**Table 3:** Average *r* ^*2*^ ± SD for adjacent SNP according to marker panel density

% SNP kept^a^	Hereford		Braford	
	Average *r* ^*2*^	Average distance (Kb)	Average *r* ^*2*^	Average distance (Kb)
50 %	0.15 ± 0.21	121.80	0.12 ± 0.17	121.83
25 %	0.11 ± 0.16	243.57	0.09 ± 0.13	243.00
20 %	0.09 ± 0.14	304.22	0.08 ± 0.11	303.78
17 %	0.09 ± 0.14	364.97	0.07 ± 0.11	364.47
14 %	0.08 ± 0.13	425.83	0.07 ± 0.10	425.30
7 %	0.07 ± 0.10	850.22	0.06 ± 0.09	849.50

^a^In relation to the total number of SNP obtained for each population after the quality control (41,207 SNP in Hereford and 41,241 SNP in Braford)

**Table 4:** Percentage of adjacent SNP with average *r* ^*2*^ > 0.2 and > 0.3 according to marker panel density

% SNP kept^a^	Hereford		Braford	
	% *r* ^*2*^ > 0.2	% *r* ^*2*^ > 0.3	% *r* ^*2*^ > 0.2	% *r* ^*2*^ > 0.3
50 %	24.49	16.41	18.33	11.09
25 %	17.35	10.59	12.52	6.69
20 %	15.02	8.33	10.12	4.96
17 %	14.80	8.03	9.98	4.58
14 %	13.18	6.59	8.95	4.03
7 %	8.37	3.83	6.76	2.70

^a^In relation to the total number of SNP obtained for each population after the quality control (41,207 SNP in Hereford and 41,241 SNP in Braford)

### Intra and inter chromosomal heterogeneity and differences between breed in *r*^*2*^

**Fig. 2:**
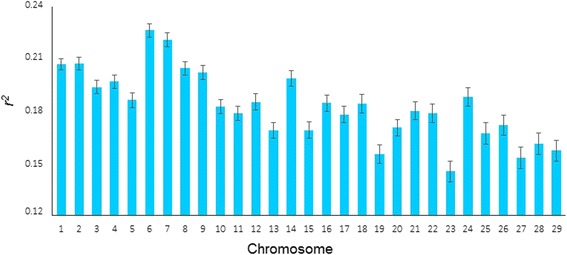
Extent of *r* ^*2*^ ± standard errors by chromosomes in Hereford and Braford populations

Average distances between SNP in different chromosomes were similar, and average physical distances between adjacent markers on Hereford and Braford autosomes was 61 Kb. For all chromosomes, average *r*^*2*^ between adjacent SNP was larger in Herefords than Brafords (Table 5). Estimated *r*^*2*^ values ranged from 0.16 (BTA23) to 0.26 (BTA6) in Herefords, and from 0.13 (BTA23, BTA28 and BTA29) to 0.20 (BTA6) in Brafords. Squared correlation estimates between adjacent SNP (Equation 2) revealed that physical distance, breed and chromosome have significant effects on *r*^*2*^ (*P* < 0.001), whereas the interaction between breed and chromosome was not significant. Therefore, predicted marginal (least square) means ± SE of *r*^*2*^ were obtained for each chromosome averaged across breeds, chromosome by breed interactions and adjacent SNP distances (Fig. 2).

**Table 5:** Average *r* ^*2*^ ± SD and mean of length distances between adjacent markers in chromosomes

Chr^a^	Length (Mb)	Hereford		Braford	
		n SNP^b^	Average *r* ^*2*^	n SNP	Average *r* ^*2*^
1	161.02	2697	0.24 ± 0.29	2699	0.17 ± 0.23
2	137.63	2197	0.23 ± 0.29	2200	0.18 ± 0.23
3	125.67	2000	0.21 ± 0.27	2001	0.17 ± 0.22
4	120.64	1987	0.23 ± 0.28	1988	0.16 ± 0.21
5	124.68	1732	0.20 ± 0.26	1734	0.16 ± 0.22
6	119.22	2066	0.26 ± 0.30	2069	0.20 ± 0.24
7	112.63	1824	0.25 ± 0.29	1824	0.19 ± 0.24
8	116.03	1958	0.23 ± 0.29	1959	0.18 ± 0.23
9	105.59	1670	0.23 ± 0.29	1671	0.17 ± 0.23
10	104.22	1760	0.21 ± 0.27	1761	0.16 ± 0.22
11	107.25	1805	0.20 ± 0.26	1806	0.16 ± 0.22
12	91.06	1342	0.21 ± 0.27	1344	0.15 ± 0.20
13	84.18	1449	0.19 ± 0.26	1449	0.15 ± 0.20
14	84.59	1471	0.23 ± 0.28	1471	0.18 ± 0.23
15	85.26	1374	0.19 ± 0.25	1374	0.15 ± 0.20
16	81.32	1314	0.21 ± 0.27	1314	0.16 ± 0.22
17	75.00	1249	0.20 ± 0.27	1250	0.15 ± 0.21
18	65.98	1036	0.21 ± 0.27	1038	0.16 ± 0.21
19	64.01	1058	0.17 ± 0.23	1059	0.14 ± 0.20
20	72.20	1264	0.19 ± 0.25	1264	0.15 ± 0.21
21	71.08	1124	0.20 ± 0.26	1124	0.16 ± 0.22
22	61.38	1021	0.20 ± 0.27	1025	0.15 ± 0.21
23	52.29	838	0.16 ± 0.23	838	0.13 ± 0.19
24	63.40	1014	0.21 ± 0.27	1018	0.16 ± 0.21
25	42.77	753	0.19 ± 0.25	755	0.15 ± 0.21
26	51.64	867	0.20 ± 0.27	867	0.14 ± 0.21
27	45.37	763	0.17 ± 0.24	764	0.14 ± 0.20
28	46.22	747	0.19 ± 0.24	747	0.13 ± 0.18
29	51.48	827	0.18 ± 0.25	828	0.13 ± 0.19

^a^Chromosomes

^b^Number of SNP

**Fig. 3:**
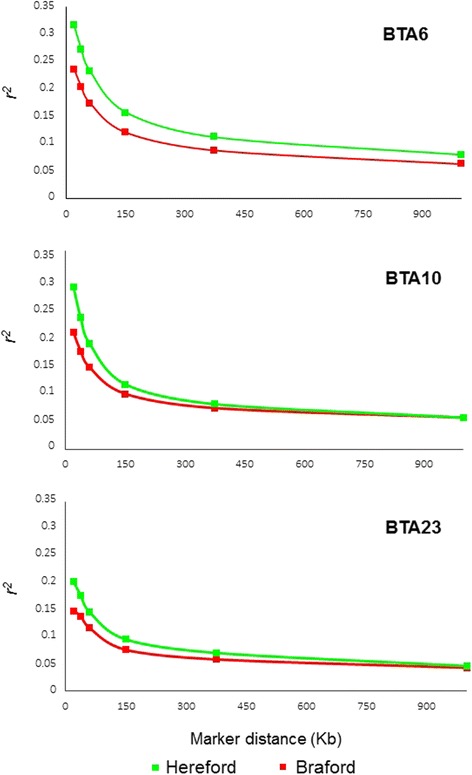
Predicted *r* ^*2*^ as a function of inter-marker distance considering different panels densities. Square symbols pinpoint predicted *r* ^*2*^ at distances of 20, 38, 60, 150, 375 and 1000 Kb corresponding, respectively, to average physical distances expected for panels of 150K, 80K, 50K, 20K, 8K, and 3K equally-spaced markers in chromosomes 6 (BTA6), 10 (BTA10) and 23 (BTA23) for Hereford and Braford populations

Squared correlation estimates considering all SNP pairs (Equation 3) revealed significant effects of linear, quadratic and cubic physical distance coefficients, breed, chromosome, and all interactions (*P* < 0.001). Predicted *r*^*2*^ values at specific distances for Herefords and Brafords are shown in Fig. 3 for chromosomes with the greatest, lowest and intermediate LD averages (BTA6, BTA23 and BTA10, respectively).

### Effective population size

**Fig. 4:**
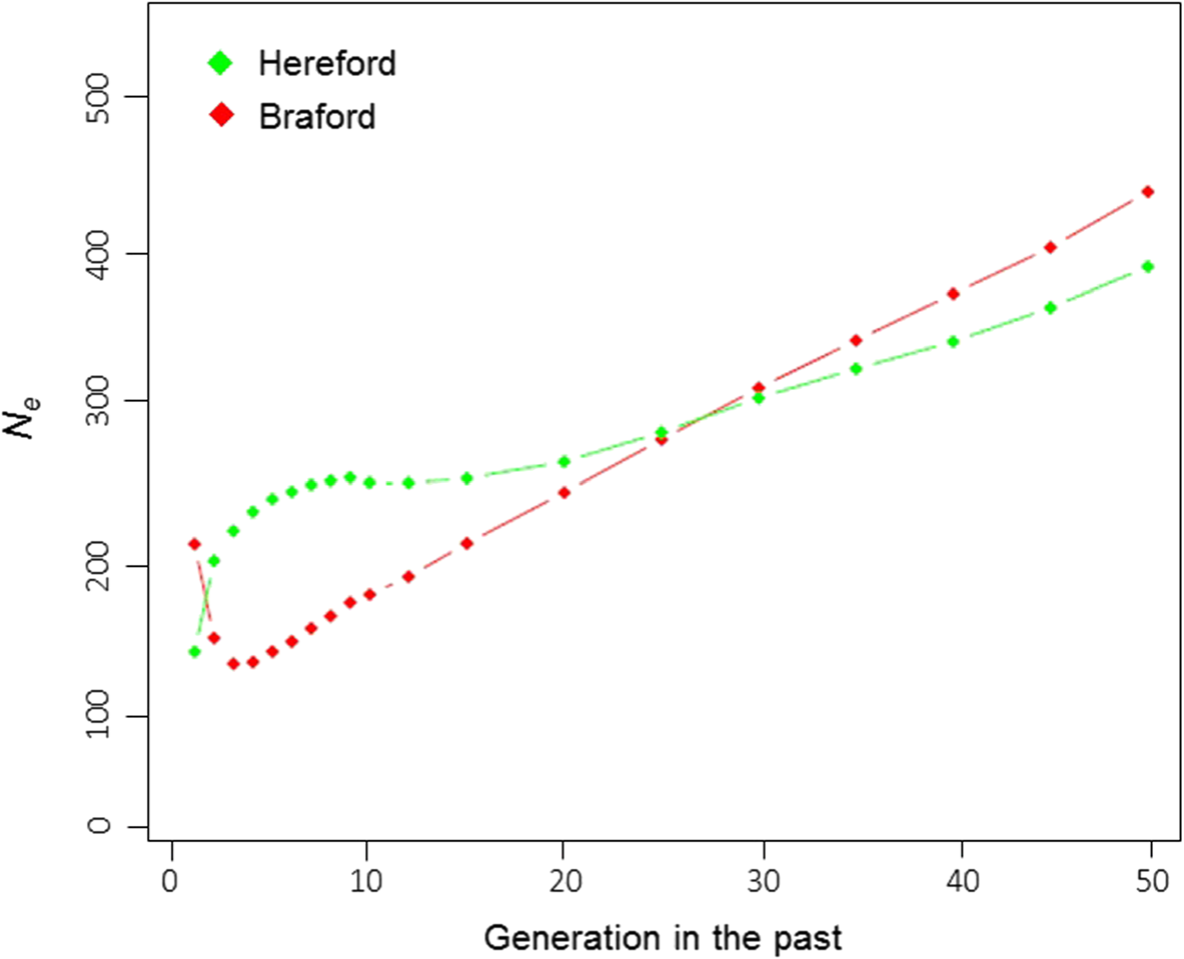
Estimated *N*_*e*_ as a function of generation in the past in Hereford and Braford populations

Although current estimated effective population size (Fig. 4) for Brafords (*N*_*e*_ = 220) is larger than for Herefords (*N*_*e*_ = 153), different recent *N*_*e*_ trends were observed for the two studied populations. Braford´s decreasing historical *N*_*e*_ trend was reversed and sharply increased in the last two generations, while Herefords had an accelerated *N*_*e*_ decline starting four generations ago. Consequently, the relative positions of the breed were changed, restoring the larger past effective size of Braford compared to Hereford, which was observed up to about thirty generations ago, when respective values were 315 and 309 for the two populations.

### Persistence of phase

**Fig. 5:**
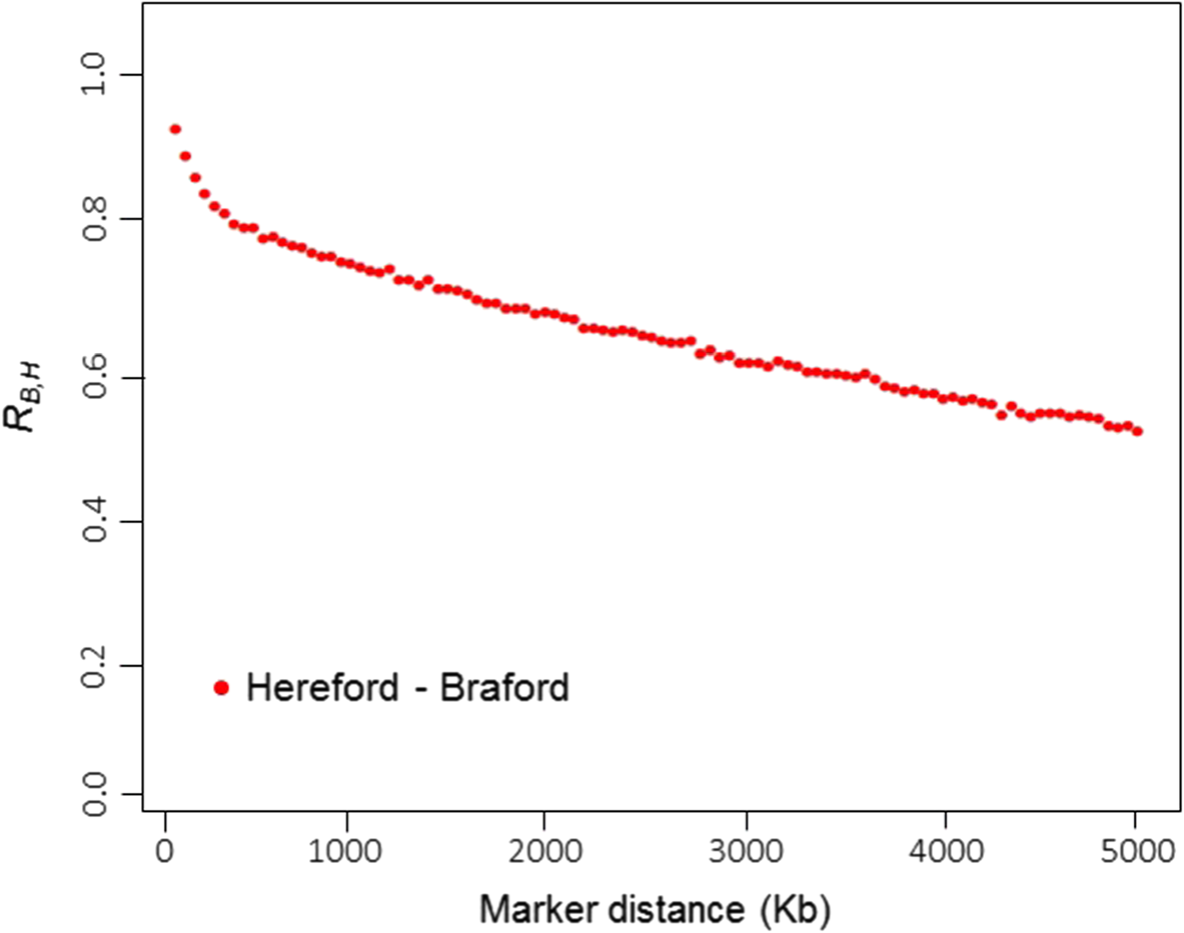
Correlation of phase (*R*_*B,H*_) between Hereford and Braford populations for SNP pairs at varying distances

Moderate to strong persistence of phase at all distances (*R*_*B*,*H*_ = 0.53 to 0.97) were observed for both breeds (Fig. 5). Phase correlations decreased rapidly with increasing distances between SNP, as was similarly observed for average *r*^*2*^. For markers < 50 Kb apart mean estimated *R*_*B*,*H*_ was 0.92, decreasing to 0.74 and 0.53 at marker distances of 1 and 5 Mb, respectively. Marker phase correlation values of *R*_*B*,*H*_ > 0.9 were found in the 0 to 50 Kb bins (*R*_*B*,*H*_ = 0.92 to 0.97), and the proportion of SNP with reversed *r* signs was lower, ranging from 5 % at 1 Kb to 34 % at 10 Mb distance.

## Discussion

Numerous studies have been conducted to estimate the pattern and extent of LD in different domestic animal species [8, 27, 28, 36]. Obtained results are essential for fine-tuning experimental designs to increase GWAS and GS efficiency and accuracies in studied populations, and can therefore have great impact in realized rates of genetic progress in economically important traits. Linkage disequilibrium patterns and scale within and between populations/breeds can be influenced by several factors such as marker allele frequencies, selection history, population structure and effective size, marker type and density, as well as which LD measure is used [9, 32, 37, 38]. Therefore, these factors should be considered when conclusions are drawn from comparisons between different studies. The choice of *r*^*2*^ [23] to estimate LD in Hereford and Braford data was based on the parameter´s lower sensitivity to variations in allele frequency [38] and sample size [39, 40], when compared to *D* and *D´*.

Robust ascertainment bias towards informativeness in taurine breeds has been observed in the 50K panel [41] and has to be considered when interpreting population genetics inferences based on LD estimates. Using a high density marker panel (446,985 SNP) and 795 genotyped Nelore steers, Espigolan et al. [42] observed an overall average *r*^*2*^ of 0.17.

Lower mean LD estimates were also reported for *B. indicus* breeds by Villa-Angulo et al. [43] when analyzing a dataset with 31,857 SNP derived from the 50K panel from 487 animals of seventeen taurine and indicine breeds. Reports of estimated larger historical effective population sizes for *B. indicus* breeds may be representative of differences which occurred during the domestication and selection processes of *B. indicus* and *B. taurus* cattle, and offers a plausible explanation for the lower LD levels observed in indicine populations [8, 44]. Considering an expected average of 3/8 Zebu contribution to Braford breed composition, the lower average *r*^*2*^ observed in this population can be a result of those differences reported for *B. indicus* and *B. taurus* breeds in terms of past effective population sizes and selection processes during breed formation.

Both studied populations presented an inverse relationship between LD and inter-marker distances, and this decline of LD as a function of distance agrees with other studies based on *r*^*2*^ estimates in cattle [8, 38, 42, 45]. The Bovine HapMap Consortium [46] reported that, in general, *B. indicus* breeds had lower *r*^*2*^ values at short distances and higher *r*^*2*^ values at longer distances between markers when compared to *B. taurus* breeds. As the extent of LD at short inter-marker distances reflect the historic effective population size [44]. Rapid decline in average *r*^*2*^ of Braford compared to the decrease of *r*^*2*^ in Hereford can be associated to differences in effective population size of the breeds.

Decreases in effective population sizes have been widely observed within the last ~50 years in several cattle breeds, mostly due to the advent of artificial insemination which allowed the intense use of fewer males and high selection pressures for specific traits [47]. As demonstrated in Fig. 4, effective population sizes for both breeds have declined over time as reflection of the historical process of domestication and breed formation [46]. Brazilian Brafords were essentially formed with a limited pool of Nelore sires and the increased *N*_*e*_ trend observed in the last two generations (Fig. 1) could have resulted from recent efforts to increase diversity of this population through the introduction of foreign lineages formed with Brahman zebu. Conversely, the decreasing *N*_*e*_ trend observed in Brazilian Herefords is supported by the known restricted use of few purebred sires observed in most recent generations (Lopa TBP, personal communication).

Moreover, selection can lead to increased interchromosomal LD heterogeneity [48]. Thus, higher LD values observed in BTA6 in Brafords and Herefords in comparison to other chromosomes can be indicative of the presence of QTL affecting traits that have been under intense selection in both breeds. Evidence of QTL in BTA6 affecting growth traits such as birth weight [49], carcass weight [50] and ribeye area [49], and also feed intake and body weight gain [51, 52], have been reported in different cattle breeds. Additionally, the “spotted” locus (*S*) [53] is also located at BTA6 in the region containing the *KIT* gene. The *S*^*H*^ allele is responsible for Hereford pattern of coat colour (white face, belly, feet and tail, often with a white stripe over the shoulder when homozygous) [53, 54]. Since the breed is fixed for this phenotype, it is expected that a historical selection occurred during breed formation fixed the spotted allele at this locus. The absence of breed × chromosome interaction effects obtained by Equation 2 indicates that even though high levels of interchromosomal LD heterogeneity were observed, similar trends were observed in Brafords and Herefords between adjacent markers at the 50K panel distances. This is in agreement with the common selection objectives applied to both populations analysed in this study [21] and to fact that Herefords are expected to have contributed 5/8 of the Braford genome. Nevertheless, as we considered a wider range of distances and had additional statistical power by including all pairs of syntenic markers in Equation 3, we were able to capture significant differences between breeds that were specific for chromosome and distance.

Knowledge about breed and chromosome-specific variation in LD levels could be used to determine optimal marker density for performing GWAS and GS studies [39, 48]. This information could be used to design customized marker panels for Hereford and Braford cattle with different chromosome-specific densities or to choose commercially available panels that would yield the desired LD levels across the whole genome. For example, if a minimal LD value of *r*^*2*^ = 0.2 is established as a threshold and the 80K panel will be used for genotyping, only BTA6 and BTA10 in Hereford and BTA6 in Braford would be expected to meet the target LD threshold (Fig. 3). To reach average *r*^*2*^ > 0.2 in all chromosomes, genotyping with 150K is required for Herefords, while for Brafords even higher densities would be required, such as available with HD commercial panels (as Illumina High-Density Bovine BeadChip Array Illumina 777K or Affymetrix Axiom Genome-Wide BOS 1 Array 650K). Alternatively, animals genotyped with panels below the desirable density could be imputed to higher densities, provided that reference haplotype panels are available for the respective populations [55].

The across-population, or across-breed, accuracy of GS estimates based on prediction equations derived from a specific reference population depends basically on the persistence of LD phase across populations, which reflects their genetic relationships [56]. Even if *r*^*2*^ values close to 1 are observed across populations, if a large proportion of SNP are in reversed phase the result of selection for these markers will lead to antagonistic responses [12]. Our estimates of phase correlation indicated that markers in LD at distances lower than 50 Kb in Herefords show similar levels of LD in Brafords, and a high proportion of these SNP share the same linkage phase. These results could be expected, since Brafords are composites with a contribution of 62.5 % of the Hereford breed. The correlation of phase values between populations proportionally decreases with the extent of divergence between the populations. To find markers that are in LD with QTL across diverging breeds, such as Australian Angus and New Zealand Jersey populations, de Roos et al. [12] concluded that a panel of approximately 300,000 marker would be required. In our case, due to the genetic similarity of Hereford and Braford breeds, 50K panel data can be pooled into a single reference population for performing GS and GWAS studies and results can subsequently be applied to either breed.

## Conclusions

Our results indicate that at least 50K and 150K of evenly spaced SNP are necessary to effectively perform GWAS and GS studies, in Hereford and Braford respectively. For distances < 50 Kb, SNP are expected to be linked to the same QTL alleles in both breeds, due to high persistence of phase; therefore, Hereford and Braford data can be pooled into a single reference population for multibreed GS and GWAS studies performed with the above mentioned marker densities.

## Ethics approval and consent to participate

Biological samples used for genotyping were collected according the Brazilian Guidelines for Use of Animals for Scientific and Educational Purposes, available at http://www.mct.gov.br/upd_blob/0234/234054.pdf.

There was a written informed consent from cattle owners (Embrapa SAIC 10200.10/01623) to cover the use of their animals and sample collection.

## Abbreviations

B: Braford
BTA: *Bos taurus* autosome
GS: Genomic selection
GWAS: Genome-wide association study
H: Hereford
HD: High density
HWE: Hardy-Weinberg equilibrium
LD: Linkage disequilibrium
MAF: Minor allele frequency
QC: Quality control
QTL: Quantitative trait loci
SD: Standard deviation
SE: Standard error
SNP: Single nucleotide polymorphisms
